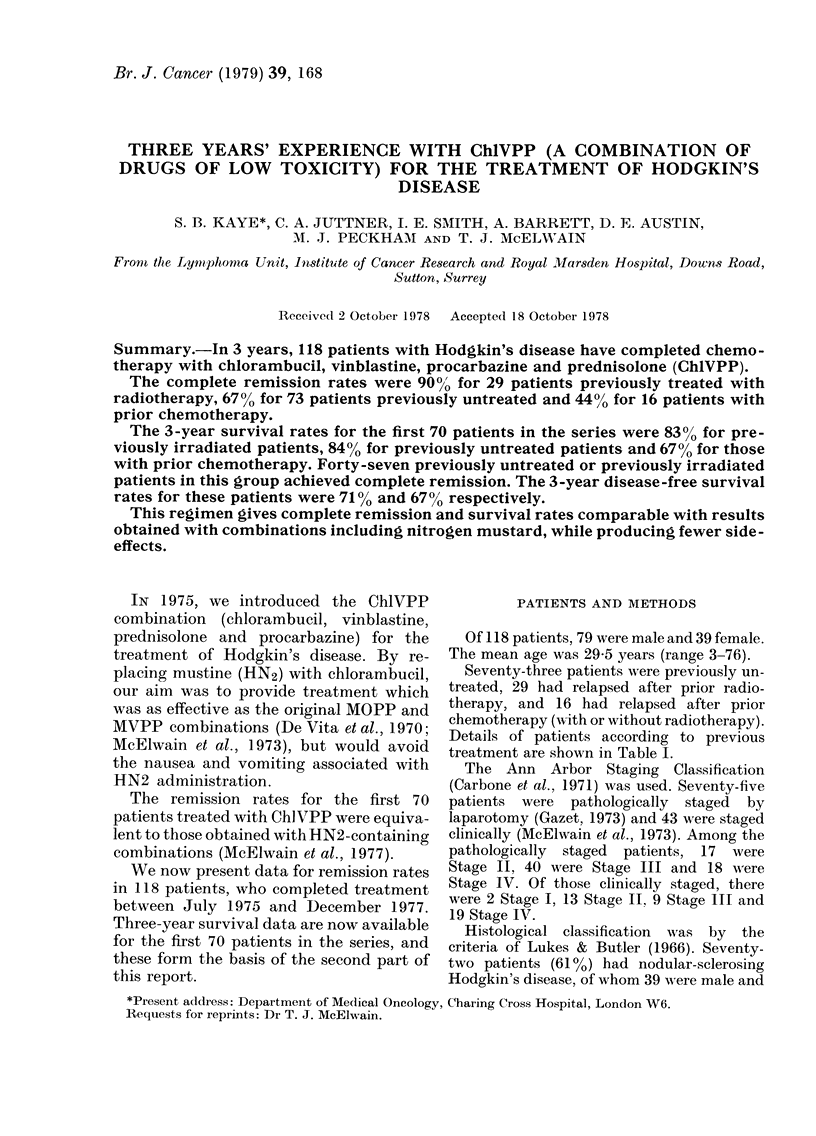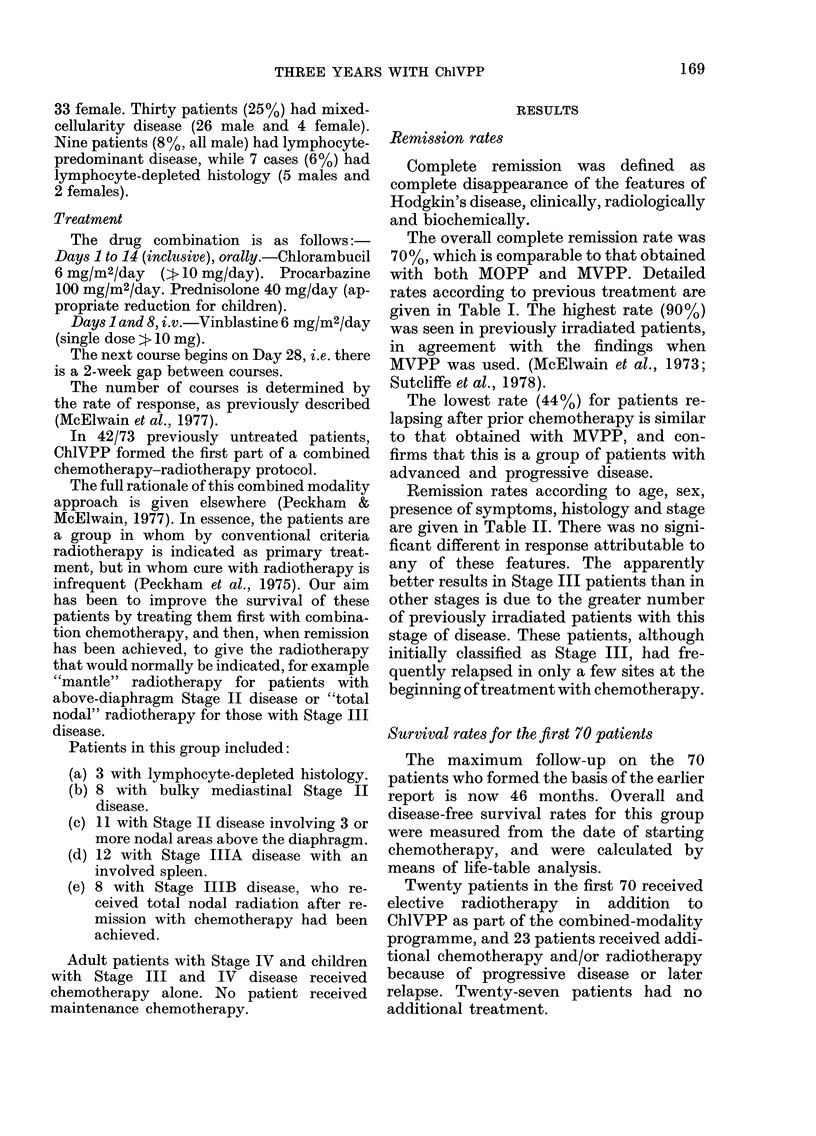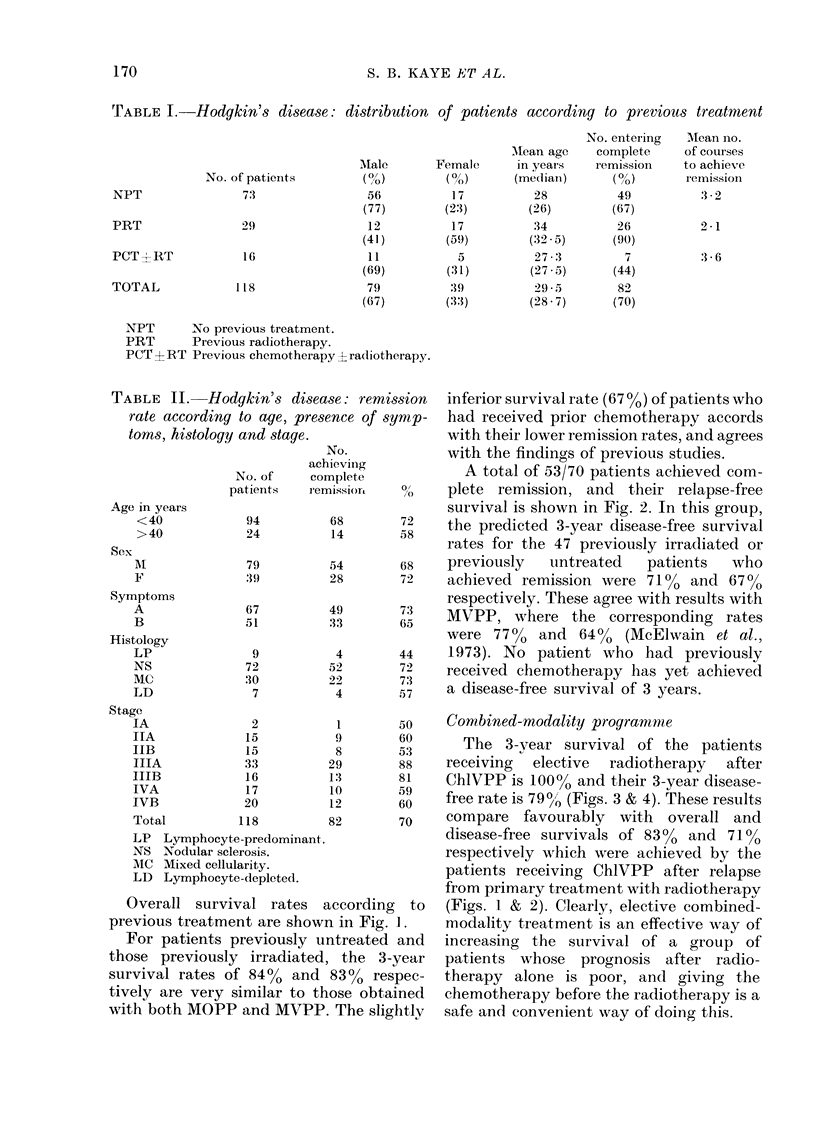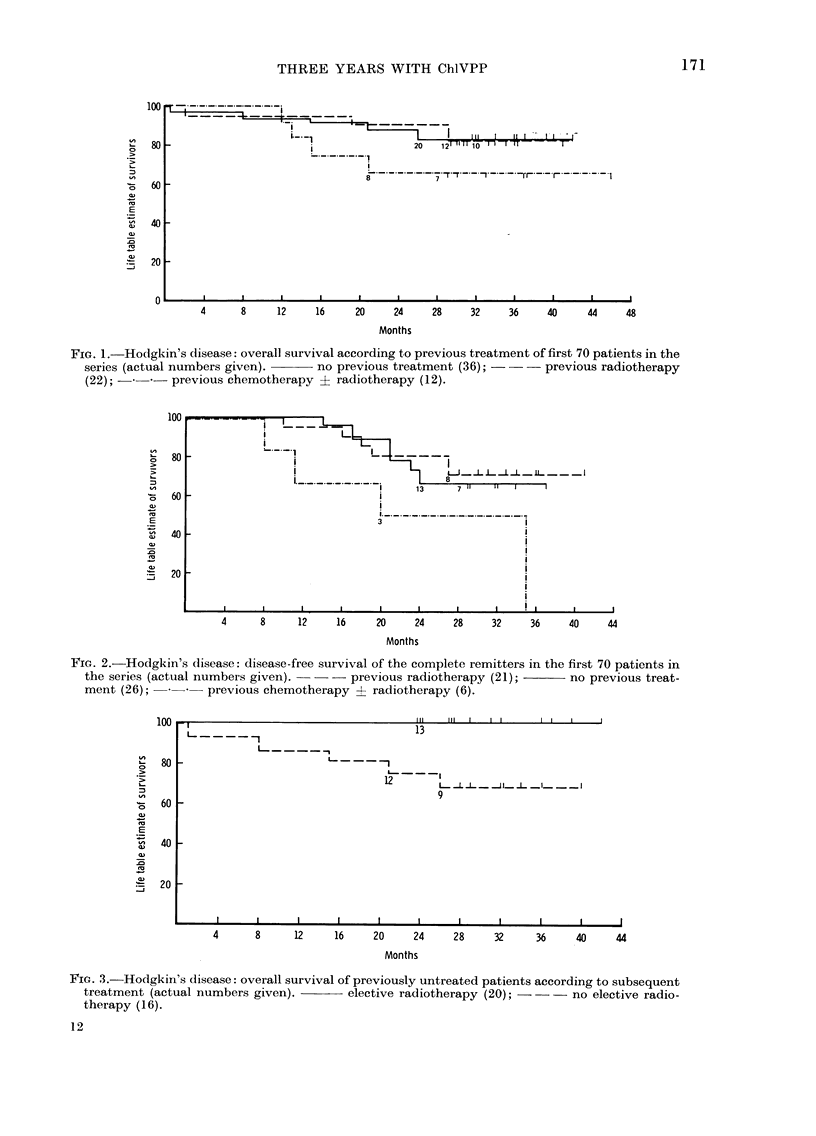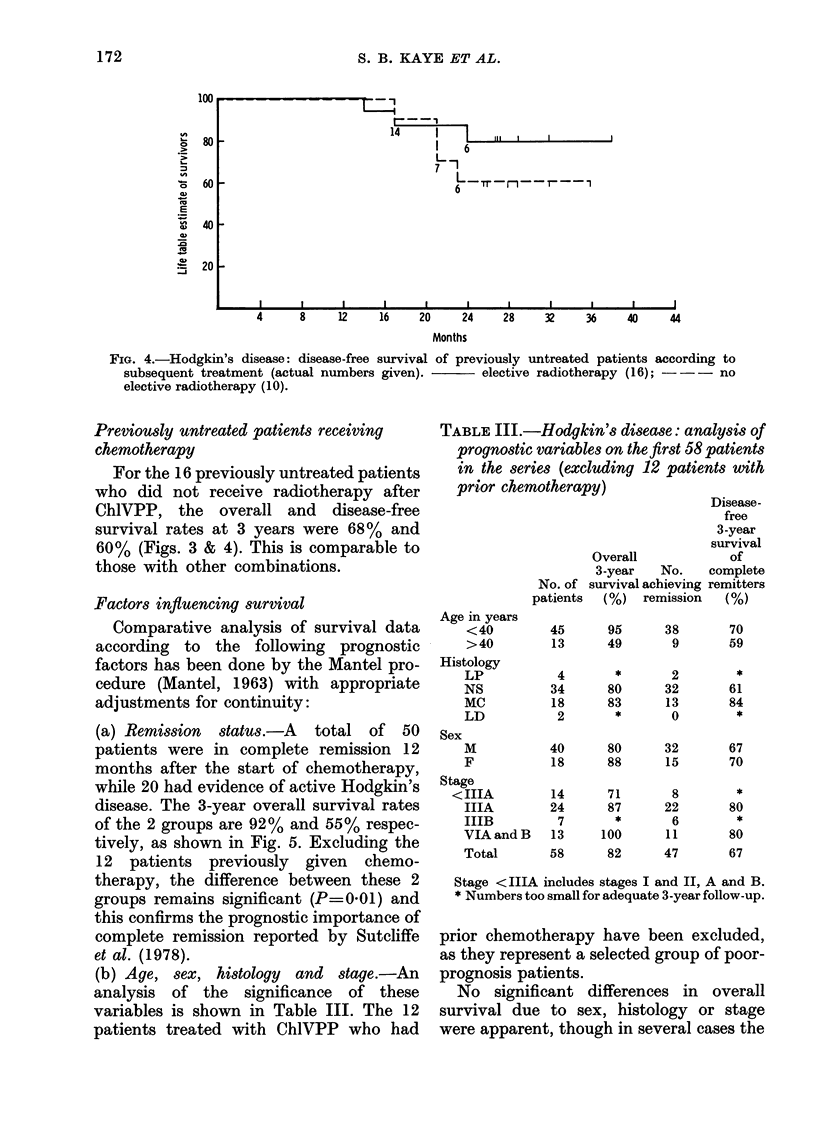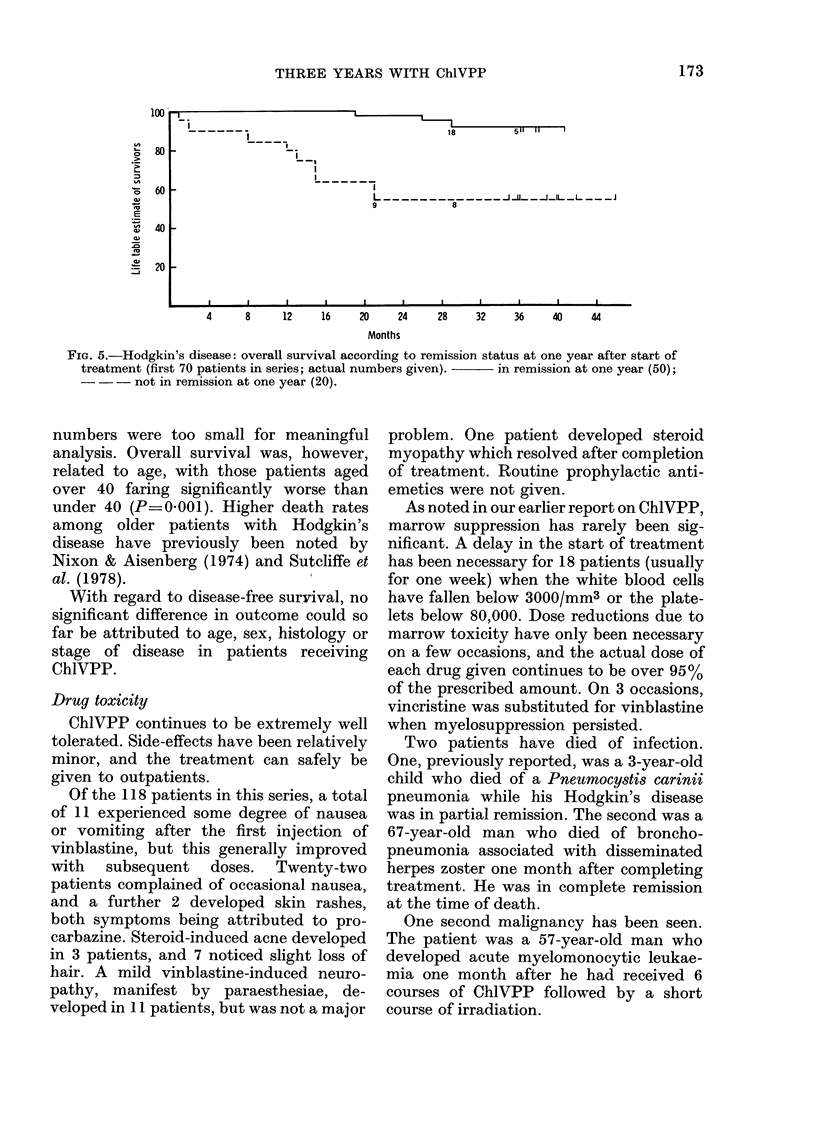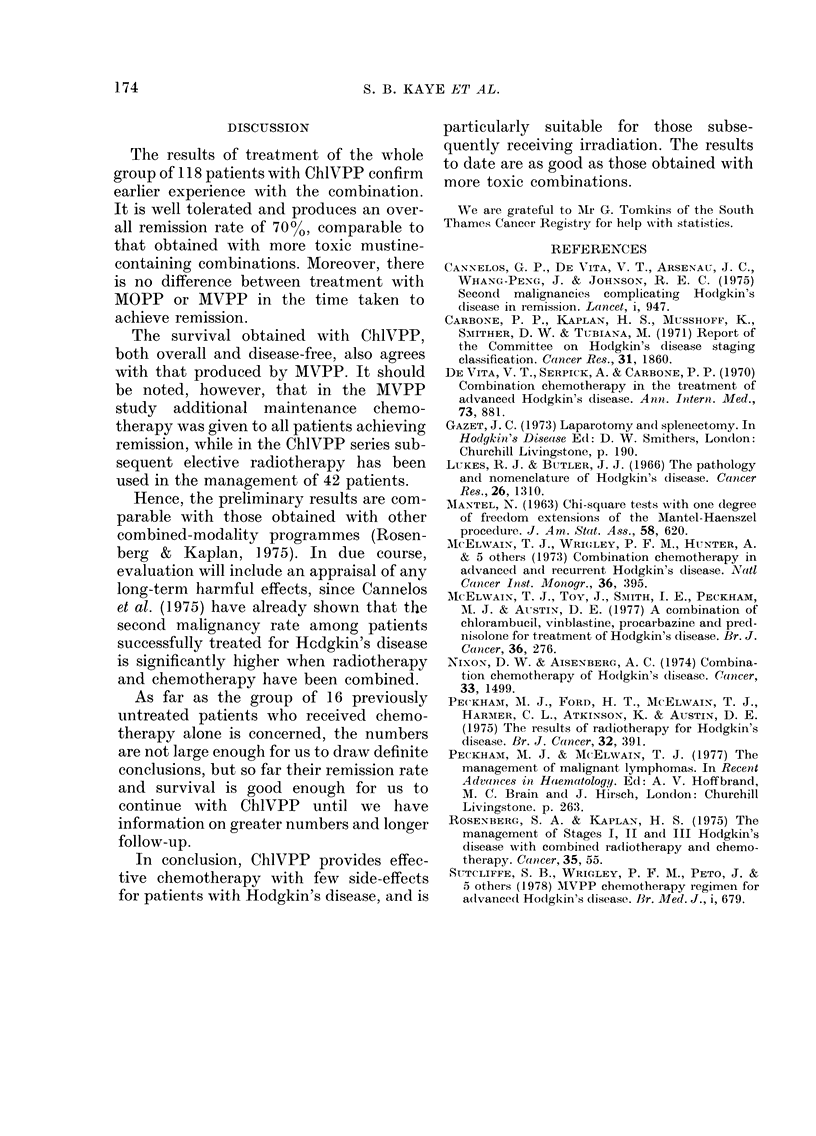# Three years' experience with Ch1VPP (a combination of drugs of low toxicity) for the treatment of Hodgkin's disease.

**DOI:** 10.1038/bjc.1979.27

**Published:** 1979-02

**Authors:** S. B. Kaye, C. A. Juttner, I. E. Smith, A. Barrett, D. E. Austin, M. J. Peckham, T. J. McElwain

## Abstract

In 3 years, 118 patients with Hodgkin's disease have completed chemotherapy with chlorambucil, vinblastine, procarbazine and prednisolone (Ch1VPP). The complete remission rates were 90% for 29 patients previously treated with radiotherapy, 67% for 73 patients previously untreated and 44% for 16 patients with prior chemotherapy. The 3-year survival rates for the first 70 patients in the series were 83% for previously irradiated patients, 84% for previously untreated patients and 67% for those with prior chemotherapy. Forty-seven previously untreated or previously irradiated patients in this group achieved complete remission. The 3-year disease-free survival rates for these patients were 71% and 67% respectively. This regimen gives complete remission and survival rates comparable with results obtained with combinations including nitrogen mustard, while producing fewer side-effects.


*Br. J. Cancer* (1979) **39**, 168

### THREE YEARS' EXPERIENCE WITH ChlVPP (A COMBINATION OF DRUGS OF LOW TOXICITY) FOR THE TREATMENT OF HODGKIN'S DISEASE

S. B. KAYE*, C. A. JUTTNER, I. E. SMITH, A. BARRETT, D. E. AUSTIN, M. J. PECKHAM AND T. J. McELWAIN

*From the Lymphoma Unit, Institute of Cancer Research and Royal Marsden Hospital, Downs Road, Sutton, Surrey*

Received 2 October 1978 Accepted 18 October 1978

**Summary.—In 3 years, 118 patients with Hodgkin's disease have completed chemotherapy with chlorambucil, vinblastine, procarbazine and prednisolone (ChlVPP).**

**The complete remission rates were 90% for 29 patients previously treated with radiotherapy, 67% for 73 patients previously untreated and 44% for 16 patients with prior chemotherapy.**

**The 3-year survival rates for the first 70 patients in the series were 83% for previously irradiated patients, 84% for previously untreated patients and 67% for those with prior chemotherapy. Forty-seven previously untreated or previously irradiated patients in this group achieved complete remission. The 3-year disease-free survival rates for these patients were 71% and 67% respectively.**

**This regimen gives complete remission and survival rates comparable with results obtained with combinations including nitrogen mustard, while producing fewer side-effects.**

IN 1975, we introduced the ChlVPP combination (chlorambucil, vinblastine, prednisolone and procarbazine) for the treatment of Hodgkin's disease. By replacing mustine ($HN_2$) with chlorambucil, our aim was to provide treatment which was as effective as the original MOPP and MVPP combinations (De Vita *et al.*, 1970; McElwain *et al.*, 1973), but would avoid the nausea and vomiting associated with HN2 administration.

The remission rates for the first 70 patients treated with ChlVPP were equivalent to those obtained with HN2-containing combinations (McElwain *et al.*, 1977).

We now present data for remission rates in 118 patients, who completed treatment between July 1975 and December 1977. Three-year survival data are now available for the first 70 patients in the series, and these form the basis of the second part of this report.

### PATIENTS AND METHODS

Of 118 patients, 79 were male and 39 female. The mean age was 29·5 years (range 3–76).

Seventy-three patients were previously untreated, 29 had relapsed after prior radiotherapy, and 16 had relapsed after prior chemotherapy (with or without radiotherapy). Details of patients according to previous treatment are shown in Table I.

The Ann Arbor Staging Classification (Carbone *et al.*, 1971) was used. Seventy-five patients were pathologically staged by laparotomy (Gazet, 1973) and 43 were staged clinically (McElwain *et al.*, 1973). Among the pathologically staged patients, 17 were Stage II, 40 were Stage III and 18 were Stage IV. Of those clinically staged, there were 2 Stage I, 13 Stage II, 9 Stage III and 19 Stage IV.

Histological classification was by the criteria of Lukes & Butler (1966). Seventy-two patients (61%) had nodular-sclerosing Hodgkin's disease, of whom 39 were male and

*Present address: Department of Medical Oncology, Charing Cross Hospital, London W6.
Requests for reprints: Dr T. J. McElwain.

33 female. Thirty patients (25%) had mixed-cellularity disease (26 male and 4 female). Nine patients (8%, all male) had lymphocyte-predominant disease, while 7 cases (6%) had lymphocyte-depleted histology (5 males and 2 females).

*Treatment*

The drug combination is as follows:—
*Days 1 to 14* (*inclusive*), *orally*.—Chlorambucil 6 mg/m$^2$/day ($\not>$10 mg/day). Procarbazine 100 mg/m$^2$/day. Prednisolone 40 mg/day (appropriate reduction for children).

*Days 1 and 8*, *i.v.*—Vinblastine 6 mg/m$^2$/day (single dose $\not>$10 mg).

The next course begins on Day 28, *i.e.* there is a 2-week gap between courses.

The number of courses is determined by the rate of response, as previously described (McElwain *et al.*, 1977).

In 42/73 previously untreated patients, ChlVPP formed the first part of a combined chemotherapy–radiotherapy protocol.

The full rationale of this combined modality approach is given elsewhere (Peckham & McElwain, 1977). In essence, the patients are a group in whom by conventional criteria radiotherapy is indicated as primary treatment, but in whom cure with radiotherapy is infrequent (Peckham *et al.*, 1975). Our aim has been to improve the survival of these patients by treating them first with combination chemotherapy, and then, when remission has been achieved, to give the radiotherapy that would normally be indicated, for example "mantle" radiotherapy for patients with above-diaphragm Stage II disease or "total nodal" radiotherapy for those with Stage III disease.

Patients in this group included:

(a) 3 with lymphocyte-depleted histology.
(b) 8 with bulky mediastinal Stage II disease.
(c) 11 with Stage II disease involving 3 or more nodal areas above the diaphragm.
(d) 12 with Stage IIIA disease with an involved spleen.
(e) 8 with Stage IIIB disease, who received total nodal radiation after remission with chemotherapy had been achieved.

Adult patients with Stage IV and children with Stage III and IV disease received chemotherapy alone. No patient received maintenance chemotherapy.

RESULTS

*Remission rates*

Complete remission was defined as complete disappearance of the features of Hodgkin's disease, clinically, radiologically and biochemically.

The overall complete remission rate was 70%, which is comparable to that obtained with both MOPP and MVPP. Detailed rates according to previous treatment are given in Table I. The highest rate (90%) was seen in previously irradiated patients, in agreement with the findings when MVPP was used. (McElwain *et al.*, 1973; Sutcliffe *et al.*, 1978).

The lowest rate (44%) for patients relapsing after prior chemotherapy is similar to that obtained with MVPP, and confirms that this is a group of patients with advanced and progressive disease.

Remission rates according to age, sex, presence of symptoms, histology and stage are given in Table II. There was no significant different in response attributable to any of these features. The apparently better results in Stage III patients than in other stages is due to the greater number of previously irradiated patients with this stage of disease. These patients, although initially classified as Stage III, had frequently relapsed in only a few sites at the beginning of treatment with chemotherapy.

*Survival rates for the first 70 patients*

The maximum follow-up on the 70 patients who formed the basis of the earlier report is now 46 months. Overall and disease-free survival rates for this group were measured from the date of starting chemotherapy, and were calculated by means of life-table analysis.

Twenty patients in the first 70 received elective radiotherapy in addition to ChlVPP as part of the combined-modality programme, and 23 patients received additional chemotherapy and/or radiotherapy because of progressive disease or later relapse. Twenty-seven patients had no additional treatment.

TABLE I.—*Hodgkin's disease: distribution of patients according to previous treatment*

| | No. of patients | Male (%) | Female (%) | Mean age in years (median) | No. entering complete remission (%) | Mean no. of courses to achieve remission |
|---|---|---|---|---|---|---|
| NPT | 73 | 56 (77) | 17 (23) | 28 (26) | 49 (67) | 3·2 |
| PRT | 29 | 12 (41) | 17 (59) | 34 (32·5) | 26 (90) | 2·1 |
| PCT±RT | 16 | 11 (69) | 5 (31) | 27·3 (27·5) | 7 (44) | 3·6 |
| TOTAL | 118 | 79 (67) | 39 (33) | 29·5 (28·7) | 82 (70) | |

NPT No previous treatment.
PRT Previous radiotherapy.
PCT±RT Previous chemotherapy±radiotherapy.

TABLE II.—*Hodgkin's disease: remission rate according to age, presence of symptoms, histology and stage.*

| | No. of patients | No. achieving complete remission | % |
|---|---|---|---|
| Age in years | | | |
| <40 | 94 | 68 | 72 |
| >40 | 24 | 14 | 58 |
| Sex | | | |
| M | 79 | 54 | 68 |
| F | 39 | 28 | 72 |
| Symptoms | | | |
| A | 67 | 49 | 73 |
| B | 51 | 33 | 65 |
| Histology | | | |
| LP | 9 | 4 | 44 |
| NS | 72 | 52 | 72 |
| MC | 30 | 22 | 73 |
| LD | 7 | 4 | 57 |
| Stage | | | |
| IA | 2 | 1 | 50 |
| IIA | 15 | 9 | 60 |
| IIB | 15 | 8 | 53 |
| IIIA | 33 | 29 | 88 |
| IIIB | 16 | 13 | 81 |
| IVA | 17 | 10 | 59 |
| IVB | 20 | 12 | 60 |
| Total | 118 | 82 | 70 |

LP Lymphocyte-predominant.
NS Nodular sclerosis.
MC Mixed cellularity.
LD Lymphocyte-depleted.

Overall survival rates according to previous treatment are shown in Fig. 1.

For patients previously untreated and those previously irradiated, the 3-year survival rates of 84% and 83% respectively are very similar to those obtained with both MOPP and MVPP. The slightly inferior survival rate (67%) of patients who had received prior chemotherapy accords with their lower remission rates, and agrees with the findings of previous studies.

A total of 53/70 patients achieved complete remission, and their relapse-free survival is shown in Fig. 2. In this group, the predicted 3-year disease-free survival rates for the 47 previously irradiated or previously untreated patients who achieved remission were 71% and 67% respectively. These agree with results with MVPP, where the corresponding rates were 77% and 64% (McElwain *et al.*, 1973). No patient who had previously received chemotherapy has yet achieved a disease-free survival of 3 years.

*Combined-modality programme*

The 3-year survival of the patients receiving elective radiotherapy after ChlVPP is 100% and their 3-year disease-free rate is 79% (Figs. 3 & 4). These results compare favourably with overall and disease-free survivals of 83% and 71% respectively which were achieved by the patients receiving ChlVPP after relapse from primary treatment with radiotherapy (Figs. 1 & 2). Clearly, elective combined-modality treatment is an effective way of increasing the survival of a group of patients whose prognosis after radiotherapy alone is poor, and giving the chemotherapy before the radiotherapy is a safe and convenient way of doing this.

FIG. 1.—Hodgkin's disease: overall survival according to previous treatment of first 70 patients in the series (actual numbers given). ——— no previous treatment (36); — — — previous radiotherapy (22); —·—·— previous chemotherapy ± radiotherapy (12).

FIG. 2.—Hodgkin's disease: disease-free survival of the complete remitters in the first 70 patients in the series (actual numbers given). — — — previous radiotherapy (21); ——— no previous treatment (26); —·—·— previous chemotherapy ± radiotherapy (6).

FIG. 3.—Hodgkin's disease: overall survival of previously untreated patients according to subsequent treatment (actual numbers given). ——— elective radiotherapy (20); — — — no elective radiotherapy (16).

FIG. 4.—Hodgkin's disease: disease-free survival of previously untreated patients according to subsequent treatment (actual numbers given). ——— elective radiotherapy (16); — — — no elective radiotherapy (10).

*Previously untreated patients receiving chemotherapy*

For the 16 previously untreated patients who did not receive radiotherapy after ChlVPP, the overall and disease-free survival rates at 3 years were 68% and 60% (Figs. 3 & 4). This is comparable to those with other combinations.

*Factors influencing survival*

Comparative analysis of survival data according to the following prognostic factors has been done by the Mantel procedure (Mantel, 1963) with appropriate adjustments for continuity:

(a) *Remission status.*—A total of 50 patients were in complete remission 12 months after the start of chemotherapy, while 20 had evidence of active Hodgkin's disease. The 3-year overall survival rates of the 2 groups are 92% and 55% respectively, as shown in Fig. 5. Excluding the 12 patients previously given chemotherapy, the difference between these 2 groups remains significant ($P = 0{\cdot}01$) and this confirms the prognostic importance of complete remission reported by Sutcliffe *et al.* (1978).

(b) *Age, sex, histology and stage.*—An analysis of the significance of these variables is shown in Table III. The 12 patients treated with ChlVPP who had

TABLE III.—*Hodgkin's disease: analysis of prognostic variables on the first 58 patients in the series (excluding 12 patients with prior chemotherapy)*

| | No. of patients | Overall 3-year survival (%) | No. achieving remission | Disease-free 3-year survival of complete remitters (%) |
|---|---|---|---|---|
| Age in years | | | | |
| <40 | 45 | 95 | 38 | 70 |
| >40 | 13 | 49 | 9 | 59 |
| Histology | | | | |
| LP | 4 | * | 2 | * |
| NS | 34 | 80 | 32 | 61 |
| MC | 18 | 83 | 13 | 84 |
| LD | 2 | * | 0 | * |
| Sex | | | | |
| M | 40 | 80 | 32 | 67 |
| F | 18 | 88 | 15 | 70 |
| Stage | | | | |
| <IIIA | 14 | 71 | 8 | * |
| IIIA | 24 | 87 | 22 | 80 |
| IIIB | 7 | * | 6 | * |
| VIA and B | 13 | 100 | 11 | 80 |
| Total | 58 | 82 | 47 | 67 |

Stage <IIIA includes stages I and II, A and B.
* Numbers too small for adequate 3-year follow-up.

prior chemotherapy have been excluded, as they represent a selected group of poor-prognosis patients.

No significant differences in overall survival due to sex, histology or stage were apparent, though in several cases the

FIG. 5.—Hodgkin's disease: overall survival according to remission status at one year after start of treatment (first 70 patients in series; actual numbers given). ——— in remission at one year (50); — — — — not in remission at one year (20).

numbers were too small for meaningful analysis. Overall survival was, however, related to age, with those patients aged over 40 faring significantly worse than under 40 ($P=0{\cdot}001$). Higher death rates among older patients with Hodgkin's disease have previously been noted by Nixon & Aisenberg (1974) and Sutcliffe *et al.* (1978).

With regard to disease-free survival, no significant difference in outcome could so far be attributed to age, sex, histology or stage of disease in patients receiving ChlVPP.

*Drug toxicity*

ChlVPP continues to be extremely well tolerated. Side-effects have been relatively minor, and the treatment can safely be given to outpatients.

Of the 118 patients in this series, a total of 11 experienced some degree of nausea or vomiting after the first injection of vinblastine, but this generally improved with subsequent doses. Twenty-two patients complained of occasional nausea, and a further 2 developed skin rashes, both symptoms being attributed to procarbazine. Steroid-induced acne developed in 3 patients, and 7 noticed slight loss of hair. A mild vinblastine-induced neuropathy, manifest by paraesthesiae, developed in 11 patients, but was not a major problem. One patient developed steroid myopathy which resolved after completion of treatment. Routine prophylactic anti-emetics were not given.

As noted in our earlier report on ChlVPP, marrow suppression has rarely been significant. A delay in the start of treatment has been necessary for 18 patients (usually for one week) when the white blood cells have fallen below 3000/mm$^3$ or the platelets below 80,000. Dose reductions due to marrow toxicity have only been necessary on a few occasions, and the actual dose of each drug given continues to be over 95% of the prescribed amount. On 3 occasions, vincristine was substituted for vinblastine when myelosuppression persisted.

Two patients have died of infection. One, previously reported, was a 3-year-old child who died of a *Pneumocystis carinii* pneumonia while his Hodgkin's disease was in partial remission. The second was a 67-year-old man who died of bronchopneumonia associated with disseminated herpes zoster one month after completing treatment. He was in complete remission at the time of death.

One second malignancy has been seen. The patient was a 57-year-old man who developed acute myelomonocytic leukaemia one month after he had received 6 courses of ChlVPP followed by a short course of irradiation.

### DISCUSSION

The results of treatment of the whole group of 118 patients with ChlVPP confirm earlier experience with the combination. It is well tolerated and produces an overall remission rate of 70%, comparable to that obtained with more toxic mustine-containing combinations. Moreover, there is no difference between treatment with MOPP or MVPP in the time taken to achieve remission.

The survival obtained with ChlVPP, both overall and disease-free, also agrees with that produced by MVPP. It should be noted, however, that in the MVPP study additional maintenance chemotherapy was given to all patients achieving remission, while in the ChlVPP series subsequent elective radiotherapy has been used in the management of 42 patients.

Hence, the preliminary results are comparable with those obtained with other combined-modality programmes (Rosenberg & Kaplan, 1975). In due course, evaluation will include an appraisal of any long-term harmful effects, since Cannelos *et al.* (1975) have already shown that the second malignancy rate among patients successfully treated for Hodgkin's disease is significantly higher when radiotherapy and chemotherapy have been combined.

As far as the group of 16 previously untreated patients who received chemotherapy alone is concerned, the numbers are not large enough for us to draw definite conclusions, but so far their remission rate and survival is good enough for us to continue with ChlVPP until we have information on greater numbers and longer follow-up.

In conclusion, ChlVPP provides effective chemotherapy with few side-effects for patients with Hodgkin's disease, and is particularly suitable for those subsequently receiving irradiation. The results to date are as good as those obtained with more toxic combinations.

We are grateful to Mr G. Tomkins of the South Thames Cancer Registry for help with statistics.

### REFERENCES

CANNELOS, G. P., DE VITA, V. T., ARSENAU, J. C., WHANG-PENG, J. & JOHNSON, R. E. C. (1975) Second malignancies complicating Hodgkin's disease in remission. *Lancet*, i, 947.

CARBONE, P. P., KAPLAN, H. S., MUSSHOFF, K., SMITHER, D. W. & TUBIANA, M. (1971) Report of the Committee on Hodgkin's disease staging classification. *Cancer Res.*, **31**, 1860.

DE VITA, V. T., SERPICK, A. & CARBONE, P. P. (1970) Combination chemotherapy in the treatment of advanced Hodgkin's disease. *Ann. Intern. Med.*, **73**, 881.

GAZET, J. C. (1973) Laparotomy and splenectomy. In *Hodgkin's Disease* Ed: D. W. Smithers, London: Churchill Livingstone, p. 190.

LUKES, R. J. & BUTLER, J. J. (1966) The pathology and nomenclature of Hodgkin's disease. *Cancer Res.*, **26**, 1310.

MANTEL, N. (1963) Chi-square tests with one degree of freedom extensions of the Mantel-Haenszel procedure. *J. Am. Stat. Ass.*, **58**, 620.

MCELWAIN, T. J., WRIGLEY, P. F. M., HUNTER, A. & 5 others (1973) Combination chemotherapy in advanced and recurrent Hodgkin's disease. *Natl Cancer Inst. Monogr.*, **36**, 395.

MCELWAIN, T. J., TOY, J., SMITH, I. E., PECKHAM, M. J. & AUSTIN, D. E. (1977) A combination of chlorambucil, vinblastine, procarbazine and prednisolone for treatment of Hodgkin's disease. *Br. J. Cancer*, **36**, 276.

NIXON, D. W. & AISENBERG, A. C. (1974) Combination chemotherapy of Hodgkin's disease. *Cancer*, **33**, 1499.

PECKHAM, M. J., FORD, H. T., MCELWAIN, T. J., HARMER, C. L., ATKINSON, K. & AUSTIN, D. E. (1975) The results of radiotherapy for Hodgkin's disease. *Br. J. Cancer*, **32**, 391.

PECKHAM, M. J. & MCELWAIN, T. J. (1977) The management of malignant lymphomas. In *Recent Advances in Haematology*. Ed: A. V. Hoffbrand, M. C. Brain and J. Hirsch, London: Churchill Livingstone. p. 263.

ROSENBERG, S. A. & KAPLAN, H. S. (1975) The management of Stages I, II and III Hodgkin's disease with combined radiotherapy and chemotherapy. *Cancer*, **35**, 55.

SUTCLIFFE, S. B., WRIGLEY, P. F. M., PETO, J. & 5 others (1978) MVPP chemotherapy regimen for advanced Hodgkin's disease. *Br. Med. J.*, i, 679.